# Severe Transaminitis in a Patient With Influenza: A Case Report

**DOI:** 10.7759/cureus.101449

**Published:** 2026-01-13

**Authors:** Daniel Rossie, Emmelyn Samones, Deena Bengiamin

**Affiliations:** 1 Emergency Medicine, Loma Linda University Medical Center, Loma Linda, USA

**Keywords:** hepatitis, influenza, rhabdomyolysis, transaminitis, viral syndrome

## Abstract

We present a case of a patient with significant liver function test (LFT) elevation and rhabdomyolysis in the setting of influenza infection. Patients with influenza infection and transaminitis have a higher mortality rate. There is limited data on the incidence of this complication, as routine laboratory values are not obtained in the emergency department for viral syndromes. Our patient had multiple reasons for transaminitis, including rhabdomyolysis, influenza infection, and possible acetaminophen toxicity. Our case report brings to light a potential complication of influenza infection, transaminitis, and the need for further evaluation to determine its clinical significance.

## Introduction

Seasonal influenza most often presents with upper respiratory symptoms, but extrapulmonary complications, such as hepatic dysfunction, may be underrecognized. While liver enzyme elevation is typically associated with hepatotropic viruses, cases of influenza-induced transaminitis, especially in the setting of rhabdomyolysis, are increasingly reported. Elevated aspartate aminotransferase (AST) and alanine aminotransferase (ALT) levels can occur in over 20% of hospitalized influenza patients, often correlating with greater illness severity [1]. Additionally, newer case studies emphasize the need for heightened suspicion of hepatic injury in patients with myalgias and recent viral illness, particularly when accompanied by elevated creatinine kinase levels and renal involvement [2]. In emergency medicine, where labs are often deferred for presumed viral syndromes, these case studies suggest that select patients may benefit from evaluation for transaminitis in cases of moderate-to-severe influenza.

## Case presentation

A 32-year-old man with no significant past medical history presented to the emergency department for lower back pain, radiating to his upper thighs, worsening over the previous week. The patient had symptoms of cough, congestion, and mild fevers one to two weeks prior to presentation, which resolved. He was managing his symptoms with over-the-counter cold medications, including acetaminophen. Initial vital signs were a heart rate of 117 BPM, blood pressure of 169/118 mmHg, temperature of 98.2°F, and 99% oxygen saturation on room air. He appeared ill on exam, with muscular tenderness in his thighs and lower back, some epigastric tenderness to palpation, and dry mucous membranes. He was found to have transaminitis, with an AST of 2,503 units per liter (U/L) (reference range 0-35 U/L) and ALT of 619 U/L (reference range 7-45 U/L). He had a normal alkaline phosphatase and total bilirubin. The patient was also found to have a creatinine phosphokinase (CPK) of 356,300 U/L (reference range 41-245 U/L) and a creatinine of 1.2 milligrams per deciliter (mg/dL) (reference range 0.7-1.3 mg/dL). Urinalysis showed large blood but no red blood cells on microscopy (Table 1). A computed tomography scan of the abdomen and pelvis, with and without contrast, was ordered to evaluate for nephrolithiasis or biliary pathology, which was unremarkable. The acute hepatitis panel was negative. The patient had an outpatient viral panel positive for influenza A two days prior to presentation.

**Table 1:** Laboratory findings in a 32-year-old male patient with influenza found to have severe transaminitis

Test Name	Observed Value	Reference Range and Units
Aspartate aminotransferase (AST) (initial)	2,503 U/L	0-35 U/L
Alanine aminotransferase (ALT) (initial)	619 U/L	7-45 U/L
Creatinine phosphokinase (CPK) (initial)	356,300 U/L	41-245 U/L
Alkaline phosphatase	58 U/L	37-132 U/L
Total bilirubin	0.2 mg/dL	0-1 mg/dL
Creatinine	1.2 mg/dL	0.7-1.3 mg/dL
Urine analysis (UA) blood	Large	Negative
UA WBC	1 hpf	0-3 hpf
AST (final)	1,667 U/L	0-35 U/L
ALT (final)	543 U/L	7-45 U/L
CPK (final)	161,740 U/L	41-245 U/L

Given the patient’s significant dehydration and rhabdomyolysis, he was admitted to the internal medicine service for hydration and renal function monitoring. He was evaluated by rheumatology while inpatient and was determined to have a low likelihood of inflammatory myopathy. Treatment for acetaminophen toxicity was considered, but the patient's acetaminophen level was normal on admission. Fulminant liver failure was also considered, but the patient's international normalized ratio was 1.2. The patient left the hospital against medical advice on hospital day 2, with a final CPK of 161,740 U/L, AST of 1,667 U/L, and ALT of 543 U/L (Table 1). 

## Discussion

There are multiple reasons for elevated liver enzymes, which include toxicological, infectious, ischemic, metabolic, malignant, and autoimmune [3]. Emergency departments have a significant number of patient visits with complaints consistent with viral upper respiratory infections, with a significant financial burden for the treatment and evaluation of these infections - roughly $40 billion annually [4]. There is no evidence at this time for routine laboratory evaluation in all of these patients; however, the question for consideration would be whether laboratory monitoring is necessary for the management and disposition of these patients.

Influenza infection has been associated with elevated liver function tests (LFTs). Specifically, “swine flu” H1N1 Influenza has a correlation with elevated LFTs in that the higher the LFTs, the greater the mortality rate [5]. Their retrospective study found no causation, but several markers, including elevated LFTs, were independent risk factors for death. The mechanism for LFT rise is unclear, but it has been suggested to be related to muscle breakdown. There is also controversy on whether the rise in LFTs is clinically significant. However, it can be suggested that patients with influenza and transaminitis, elevated to several hundred, could be considered for admission for further monitoring. 

A study showed that patients with CPK levels greater than 1,000 experienced the effect of rhabdomyolysis on LFTs [6]. Approximately 93% of patients had an elevation in their AST levels, and 75% had elevations in their ALT levels. LFTs decreased in correlation with their decreasing CPK levels, which was attributed to the AST being elevated secondary to skeletal muscle breakdown. Another study suggests that influenza-related rhabdomyolysis causes the “breakdown of muscle tissue, causing release of CPK, aldolase, lactate dehydrogenase, ALT, and AST into the blood” [7]. This is why the authors recommend prompt identification of this pathology to prevent long-term nephrotoxicity.

Our patient presented with myalgias and previously had upper respiratory symptoms. He was found to have an influenza A infection, with elevated AST, ALT, and CPK. The patient’s LFTs could have been elevated due to his significantly elevated CPK level. They could also have been elevated due to medication use. The patient was using an unknown amount of over-the-counter cold medications, some of which included acetaminophen in their formulation. Treatment for toxicity was considered, but the patient's acetaminophen level returned undetectable. Influenza is also associated with elevated LFTs and increased mortality. Our patient’s presentation and workup revealed an unusual presentation of influenza. More information, however, is needed to establish the incidence of liver injury, rhabdomyolysis, and their clinical significance in this patient population.

## Conclusions

The patient, with a confirmed influenza A infection two days earlier, presented to the emergency department with myalgias and was found to have transaminitis, rhabdomyolysis, and acute kidney injury. Patients with a viral infection, including influenza, who have laboratory findings such as elevated LFTs, CPK, and creatinine may have a possible correlation with increased mortality. However, the correlation between influenza infection and transaminitis needs further investigation. Patients with severe viral illness or symptoms can be considered for early laboratory evaluation to check for these abnormalities.
